# Effect of Fuel Preheating on Engine Characteristics of Waste Animal Fat-Oil Biodiesel in Compression Ignition Engine

**DOI:** 10.3390/polym14183896

**Published:** 2022-09-18

**Authors:** Gokul Raghavendra Srinivasan, Ranjitha Jambulingam, Amel Gacem, Akil Ahmad, Javed Khan Bhutto, Krishna Kumar Yadav, Amine Mezni, Omar Khulaif R. Alharbi, Saiful Islam, Yongtae Ahn, Byong-Hun Jeon

**Affiliations:** 1 Research and Development Department, Steamax Envirocare Private Limited, Delhi 110058, India; 2 CO_2_ Research and Green Technologies Centre, Vellore Institute of Technology, Vellore 632014, India; 3 Department of Physics, Faculty of Sciences, University 20 Août 1955, Skikda 21000, Algeria; 4 Chemistry Department, College of Science and Humanities, Prince Sattam Bin Abdulaziz University, Al-Kharj 11942, Saudi Arabia; 5 Department of Electrical Engineering, College of Engineering, King Khalid University, Abha 61421, Saudi Arabia; 6 Faculty of Science and Technology, Madhyanchal Professional University, Ratibad, Bhopal 462044, India; 7 Department of Chemistry, College of Science, Taif University, P.O. Box 11099, Taif 21944, Saudi Arabia; 8 Civil Engineering Department, College of Engineering, King Khalid University, Abha 61421, Saudi Arabia; 9 Department of Earth Resources & Environmental Engineering, Hanyang University, 222-Wangsimni-ro, Seongdong-gu, Seoul 04763, Korea

**Keywords:** animal wastes, waste animal fat-oil biodiesel, fuel preheating, engine characteristics, flow characteristics, atomization and vaporization

## Abstract

The present study aims at understanding the effects of fuel preheating on engine characteristics of waste animal fat-oil (WAF-O) biodiesel in a single-cylinder CI engine, with the preheating technique proposed as an effective means for enhancing the fuel properties. To understand the effects of the preheated fuel, the WAF-O biodiesel was preheated at 60, 80, 100 and 120 °C and tested along with neat diesel and unheated WAF-O biodiesel. For this purpose, biodiesel was produced from different animal wastes by means of KOH-assisted ethanol-based transesterification, reporting its maximum yield as 96.37 ± 1.8%, with significant distribution of unsaturated oleic acid, saturated palmitic acid and stearic acid. Upon evaluating its fuel characteristics as per ASTM D6751 standards, a rise in preheating temperature by 1 °C reduced the density and kinematic viscosity of WAF-O biodiesel by 0.383 kg/m^3^ and 0.025 mm^2^/s, respectively, and was explained by the weakening of intermolecular forces between its fatty acid ester molecules. Preheated samples reported superior combustion characteristics by exhibiting increased in-cylinder pressure (2.24%, on average) and heat release rates in addition to their shortened ignition delay (1–4 °CA). Furthermore, preheating of WAF-O biodiesel reduced its specific fuel consumption and increased its brake thermal efficiency by 7.86% (on average) and 9.23% (on average), respectively. However, higher preheating temperatures (>120 °C) resulted in increased fuel consumption owing to its varied flow characteristics. In addition to the changes in combustion characteristics, preheating WAF-O bio-diesel also resulted in reduced carbon monoxide, nitrous oxide and hydrocarbon emission by 13.88%, 7.21% and 26.94%, respectively, and increased carbon dioxide emission by 7.58%. Summing up, the enhancements in overall engine characteristics of preheated samples were accounted for by their improvised fuel injection characteristics due to their reduced density and viscosity, which ensured for their effective combustion.

## 1. Introduction

The increased usage of biodiesel as an effective alternative biofuel for both industrial and transportation sectors has made many researchers and scientists focus on producing it effectively with minimal resource utilization at a large scale. This rise in biodiesel consumption is due to its renewability, self-sustainability, and carbon neutrality, leading to enhanced engine performance under reduced emission levels. Eventually, enhanced engine characteristics from biodiesel fuel are explained by the enhanced cetane number (CN) and high fuel-bound oxygen content, in addition to zero aromatics and sulfur content [1,2].

For decades, biodiesel have been produced from a wide variety of feedstocks ranging from unsaturated oils extracted from non-edible seeds [3,4,5] to saturated fats rendered from animal wastes [6,7] and discarded waste greases [8]. In recent times, biodiesel have also been produced from waste pyrolysis oils [9] and other renewable feedstocks [10,11]. Fats rendered from animal wastes have gained attention in view of their high energy density, inedibility and ability to produce good quality biodiesel [6]. Commonly used waste animal fats (WAF) include beef tallow [12], pork lard [13], mutton suet [14], and chicken fat [15], with beef tallow being widely preferred for large-scale biodiesel production [16]. Valorizing these wastes serve as an effective measure to reduce environmental pollution and function as an ideal example of waste-to-energy conversion technique. In general, many production techniques have been developed for producing biodiesel efficiently and include traditional transesterification catalyzed by using homogeneous acid and base catalysts, ionic liquids, enzymes, and heterogeneous catalysts [3,17]. Moreover, recent advancements in biodiesel production have introduced ultrasonics-, microwave-, and electromagnetic induction-assisted heating for transesterification and have been proven to be very effective in producing high-quality biodiesel under reduced reaction time [18,19,20].

Great focus has also been given to improving the overall performance of biodiesel in compression ignition (CI) engines. Many feasible techniques have been proven to be highly effective in producing desirable engine outputs, including preheating [21], blending [22], and the introduction of additives [23] or nanoparticles [24] to the biodiesel mix prior to being introduced into the engine. However, considering the real-time scenario and volume of biodiesel consumed at large scales, techniques such as fuel preheating is regarded as a simple yet effective method for achieving desired fuel properties as required for efficient engine runs.

Supporting this, many researchers have reported using preheated biofuel in CI engines. For instance, Nicolici et al. (2018) identified WAF as a highly performing feedstock for fueling a CI engine and acknowledged this superior behavior in view of its higher CN and calorific value (CV) (slightly inferior to neat diesel). However, WAF performed poorly upon using it directly due to its very high viscosity, easily solidifiable nature and poor vaporization characteristics. Preheating WAF served as an effective measure for reducing its high viscosity and making it suitable for engine applications. In conclusion, this study proposed that preheating WAF above 40 °C makes it soluble with neat diesel and reported higher engine performance [25]. Furthermore, Cernat et al. (2019) investigated the combustion behavior of preheated raw animal fats-diesel fuel blends in a CI engine; and maintained the blend at 5% and 10% of fats with neat diesel. This study reported a reduction in the Heat Release Rate (HRR) by 16% and 20% for the 5% and 10% blends, respectively, and quoted a reduction in nitrous oxide (NO_X_) emission by 22%. In addition, smoke opacity was reduced by 22% and 52% for the 5% and 10% blends, respectively, in view of the reduction in viscosity, leading to enhanced atomization due to fuel preheating [26]. In addition, engine performance of raw *Azadirachta indica* biofuel (neem oil), preheated at 80 °C using heat from the exhaust gas, reported higher brake thermal efficiency (BTE) (by 5.5%) and reduced carbon monoxide (CO) (by 19.01%) and hydrocarbon (HC) (by 22.3%) emissions in the event of complete combustion due to their higher degree of fuel atomization and vaporization. In addition, this study recorded reduced exhaust gas temperature (EGT) (~370 °C), owing to reduced viscosity; however, they reported higher NOx emissions (by 18.6%) in view of increased adiabatic flame temperatures [27].

In recent times, biodiesel have also been preheated to reduce their viscosities further, which will eventually help in improving their overall engine performance and reducing emission levels. Supporting this, Kodate et al. (2020) carried out a study related to the engine characteristics of Karanja oil methyl ester (KOME) biodiesel, preheated at a temperature of 95 °C in a single-cylinder CI engine. Preheating of KOME biodiesel reduced its viscosity, which ensured enhanced atomization and vaporization, and resulted in better combustion, thereby improvising its overall engine performance and reducing emission levels. Accordingly, the brake-specific fuel consumption (BSFC) of preheated KOME was reduced by 6.5%, which helped to improve the BTE by 9.1% compared to unheated KOME. Likewise, harmful CO and HC emissions were reduced by 8.1% and 10.6%, respectively, thereby signifying that preheating induced complete combustion inside the cylinder. However, NOx emission remained higher for both unheated and preheated KOME in the event of their complete oxidation using their fuel-bound oxygen [21].

Likewise, Mekonen et al. (2020) studied the effect of preheating and fuel injection pressure of castor oil methyl ester (COME) and palm oil methyl ester (POME) in a CI engine along with neat diesel. Here, this study maintained 114 °C and 212 Bar as the effective preheating temperature and ideal fuel injection pressure, respectively. Preheating of the biodiesel samples reduced the density and viscosity to 855 kg/m^3^ and 5.02 mm^2^/s, respectively, for COME, and 864 kg/m^3^ and 3.74 mm^2^/s, respectively, for POME. Both preheated neat COME and POME samples exhibited enhanced performance characteristics by reporting reduced BSFC (by 48% for COME sample B100 and 44% for POME B100) and increased BTE (by 52.4% for COME B100 and 34.9% for POME B100). Augmentation of the exhaust gas system for fuel preheating improved the fuel properties of the biodiesel samples up to 114 °C but resulted in negative consequences such as fuel leakage and poor lubricity upon raising the temperature beyond 138 °C [28]. Similar results were reported by Mourad and Noureldenn, (2019) upon investigating the engine characteristics of sunflower oil biodiesel in a CI engine, preheated at 70 °C using the heat energy recovered from exhaust gas waste [29].

Bayrakceken (2011) studied the effects of preheating in deciding the engine characteristics of animal tallow biodiesel in a CI engine by preheating the biodiesel to 70 °C (sample B100-70) and to 100 °C (sample B100-100). Here, the SFC of the B100-30 sample was found to be 16.41% greater than neat diesel, while there was a reduction of 12.48% and 6.59% for the B100-70 and B100-100 samples, respectively. CO emission was reduced by 23.98%, 38.92%, and 45.77% (on average) for B100-30, B100-70, and B100-100, respectively. Conversely, a rise in preheating temperature reduced the EGT and NO_X_ emissions of the animal tallow biodiesel. In spite of these detailed results, this study failed to report the engine characteristics of animal fat biodiesel beyond 100 °C, which was later identified as a research gap for this present study [30].

Presently, most second-generation biodiesel are produced from waste feedstocks, especially from waste oil and fats, and are sourced from diversified sources and multiple feedstocks to maintain the commercial and industrial scale supply chain. Hence, these biodiesel exhibit collective characteristics corresponding to their feedstocks and can report varied properties that can challenge their effective performance in CI engines. Aimed at improving this performance, these biodiesel must undergo certain pre-treatments wherever applicable, and amongst them, preheating can be seen as feasible in practice, as discussed earlier. Thus, it becomes of prime importance to understand the engine characteristics of these preheated biodiesel and their effectiveness in satisfying the necessary energy needs. Many studies have reported the engine characteristics of preheated oil and oil-based biodiesel, yet only very limited studies have been carried out to understand the behavior of preheated fat-based biodiesel in the CI engine. To be more specific, no studies have reported on the preheating of biodiesel produced from waste animal fat-oil (WAF-O) rendered from multiple sources and different species since large-scale biodiesel applications use heterogeneous fat-based feedstocks. In addition, many studies have been carried out at a particular fuel preheating temperature, and many have failed to report variations in engine characteristics with different inlet fuel temperatures.

Considering these gaps, this present study focused on studying the engine characteristics of preheated biodiesel produced from waste fat rendered collectively from beef, pork, mutton and chicken wastes discarded from different tanneries, slaughterhouses and meat-processing plants. Studies have been carried out at different fuel inlet temperatures to understand their performance, combustion behavior and emission concentrations in the CI engine. Characterization and production of WAF-O biodiesel were also carried out, along with the evaluation of its fuel properties as per the American Society for Testing and Materials (ASTM) D6751 standards.

## 2. Materials and Methods

### 2.1. Collection and Analysis of Raw Animal Wastes

The raw feedstocks used in this present study were collected from the following commercial properties: (i) mutton and beef wastes for rendering tallow and suet fromleather tanneries (raw to wet blue processing) and slaughterhouses; (ii) pork and chicken wastes for rendering lard and chicken fat oil from slaughterhouses and meat processing plants. Following this, the collected waste samples were preserved under refrigeration at room temperature before being used for further processes. Reagents and solvents such as ethanol, orthophosphoric acid, hexane, potassium hydroxide and glycerol used during the different stages of WAF-O biodiesel production (rendering, refining and production) were procured from Sigma Aldrich chemicals.

Next up, the collected wastes were analyzed for their proximate composition in order to estimate the overall available moisture, fat, protein and ash content and were analyzed individually in triplicates as per standard Association of Official Analytical Chemists (AOAC) 2005 testing methods (moisture content: AOAC 925.10, fat content: AOAC 2003.05, ash content: AOAC 923.03, protein content: AOAC 984.13) [31]. For this purpose, 100 g of sample was tested for each individual sample, and the resultant values were reported in terms of mean value ± standard deviation.

### 2.2. Rendering and Refining of WAF-O

WAF-O was rendered from the collected beef, mutton, pork and chicken wastes using autoclave extraction based on the principle of a dry rendering technique. Accordingly, the wastes were minced into fine pieces and placed on a perforated vessel inside a steam jacketed container wherein the operating temperature and pressure were maintained at 140 °C and 2.5 bar, respectively. A high concentration of saturated steam inside the container forced out the WAF-O from these wastes, and the rendered WAF-O was collected at the bottom of the container. As part of the refining process, the rendered WAF-O was heated to 110 °C to remove any residual moisture content and was degummed using orthophosphoric acid under the influence of continuous heating for 20 min with stirring for the first 10 min to remove any phospholipids from it [6].

Post refining, the rendered WAF-O was analyzed for its fatty acids (FAs) content using gas chromatography-mass spectroscopy (GC-MS) analysis, where it was transesterified into its ester form by refluxing it with ethanol and 1–2% concentrated sulfuric acid based on the standard sample preparation technique [32]. On the other hand, the WAF-O biodiesel sample was directly characterized for its fatty acid esters (FAEs), without any necessity for sample preparation before being subjected to the GC-MS analysis.

### 2.3. Transesterification of Rendered WAF-O

**Figure 1:**
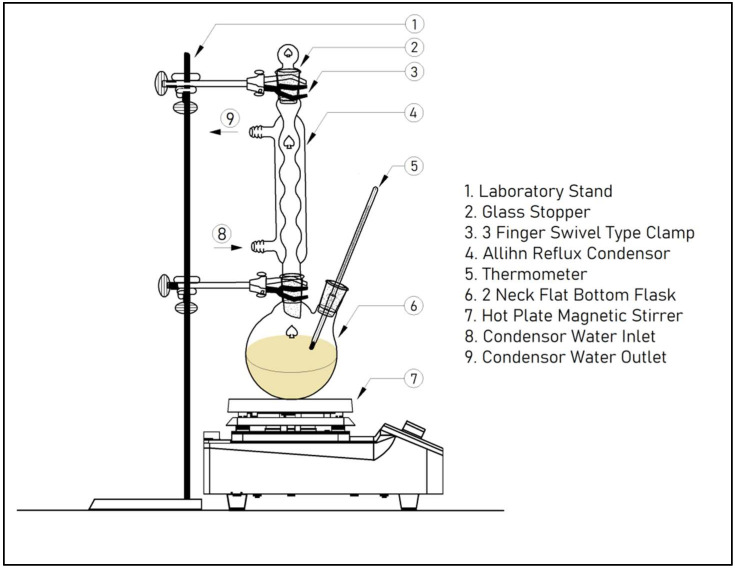
Schematic representation of the biodiesel production setup [34].

WAF-O was converted into biodiesel using ethanol-based transesterification by adding potassium hydroxide (KOH) as the homogeneous base catalyst. To begin with, the reaction was carried out in a 250 mL flat-bottomed flask mounted on a 2 L Remi hot plate magnetic stirrer and was equipped with a reflux condenser to avoid the evaporation of ethanol during higher temperature experimental runs. Figure 1 illustrates the experimental setup used for biodiesel production.

The next steps involved experimental optimization using the One Factor at a Time (OFAT) method. The necessary reaction parameters were molar ratio, catalyst concentration, and reaction temperature and time. This optimization technique involved varying one reaction parameter at a time while maintaining others as constant controls. The molar ratio varied between 1:6 and 1:12, while catalyst concentrations varied between 1 and 3 wt.%. In addition, reaction temperatures varied between 65 and 85 °C, while reaction time varied between 90 and 125 min, respectively. From the optimized molar ratio, the volume of ethanol required for transesterifying the WAF-O was calculated using Equation (1) [6].

(1) $$V_{alchol}=\frac{V_{sample}\times m\times \rho_{TG}\times M_{alchol}\;}{[92.17-3+\left[3\left(\sum_{i=1}^{n}M_{FA}\times x_{i})-17\right]\right]\times \rho_{alchol}}$$

After completion of the reaction, the synthesized biodiesel was isolated from the residual glycerol using a separating funnel, and the isolated crude biodiesel was washed with hot water progressively to remove any unreacted WAF-O or salts. Post washing, the WAF-O biodiesel was heated to 90–100 °C to remove any residual moisture content [33].

### 2.4. Evaluation of Fuel Properties of WAF-O Biodiesel

To ensure its compatibility with CI engines, the refined WAF-O biodiesel was evaluated for its fuel properties as per ASTM D6751 standards, and the results were compared with the fuel properties of neat diesel. Density and specific gravity of WAF-O biodiesel were measured as per the ASTM D1298 standard technique, using a simple BS718 series M50SP hydrometer (calibrated at 15 °C, accuracy: ±0.0006 g/mL). Kinematic viscosity of the biodiesel sample was measured using a calibrated glass-viscosity tube (accuracy: ±0.02 mm^2^/s), as per the ASTM D445 standard technique. Flash point and fire point were determined using a Pensky–Martens closed-cup apparatus, as prescribed by the ASTM D93-16 method, whereas properties such as cloud and pour point were determined using an MPP 5 gs simultaneous cloud and pour point analyzer in accordance with ASTM D2500 and D7346-15, respectively. The CN of the test sample was determined according to the ASTM D613 method, while the calorific value of WAF-O biodiesel was measured using a digital bomb calorimeter (model name: NSLI INDIA 34) by following the standards mentioned in the ASTM D240 method. Acid value and free fatty acid (FFA) content in the biodiesel sample were estimated as per the ASTM D664 standard technique. Lastly, the fundamental elements carbon, hydrogen, oxygen, phosphorous and sulfur were quantified to determine their distribution in the resultant biodiesel and were calculated per the ASTM D5291 standard technique.

### 2.5. Assessment of Engine Characteristics of WAF-O Biodiesel

**Figure 2:**
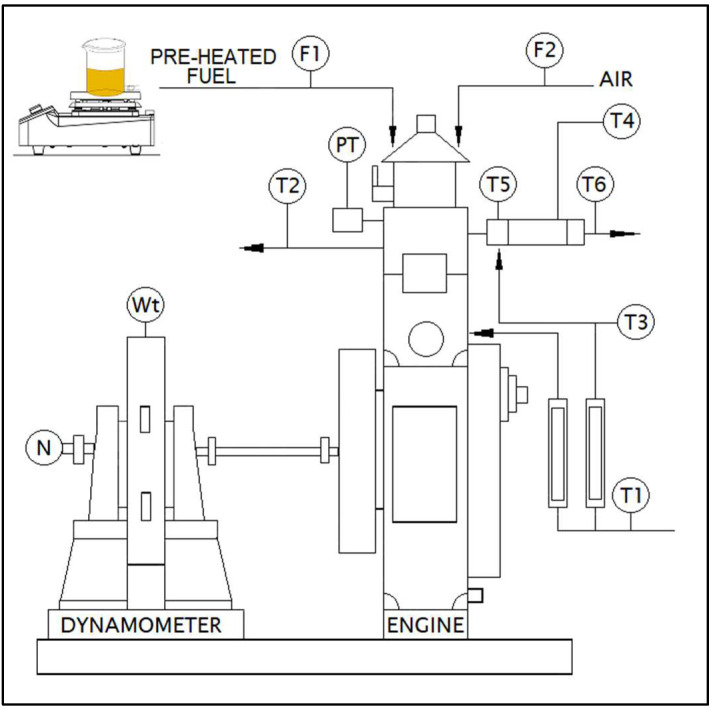
A 2D representation of test engine setup. Source: Apex Innovations Private Limited (Maharashtra, India).

The effect of preheating on engine characteristics of WAF-O biodiesel was studied by testing the preheated biodiesel samples at different fuel inlet temperatures along with neat diesel and unheated WAF-O biodiesel. Accordingly, the WAF-O biodiesel was heated at four different preheating temperatures maintained at 60, 80, 100 and 120 °C. Their corresponding samples were named as WAFOEE 60, WAFOEE 80, WAFOEE 100 and WAFOEE 120, respectively. For preheating, the WAF-O biodiesel was heated inside a cylindrical glass container with the bottom side arm mounted on a hot plate magnetic stirrer. Test samples were heated slightly above the specified temperature and were directly fed into the fuel tank for injection into the CI engine. All the test samples were injected in their neat and unblended form, and a neat diesel sample was injected into the engine after every experimental run. Subsequently, the test samples were analyzed for combustion behavior, and fuel consumption rate and emissions levels were required to assess the effect of preheating of WAF-O biodiesel on the engine. All testing was carried out in a single-cylinder Kirloskar TV1 Engine, and the recorded data were processed into desired results with the help of ICEngineSoft software developed by Apex Innovations. For instance, data related to in-cylinder pressure and heat release rate were measured with respect to the crank angle and were recorded in the form of a data sheet, which was later used to calculate the ignition delay and combustion duration of the test samples. Likewise, the rate of fuel consumption and their corresponding thermal efficiencies were calculated by correlating the time taken for the consumption of 10 cc of fuel samples with their fuel density and calorific value, along with the engine power. On the other hand, a concentration of CO, CO_2_, NOx and HC emissions in the exhaust gas were measured directly from the readings reported by the AVL DI GAS 444 N type flue gas analyzer. Figure 2 illustrates the 2D representation of the test engine setup, and Table 1 consolidates the technical specifications of the test engine and flue gas analyzer. Here, T1-T6 signifies the thermocouples connected to the processor for measuring temperatures at different points of the engine setup, which later on will be used in thermodynamic calculations. Meanwhile, fuel and air flow will be monitored using a flow meter (F1 and F2) and will be used to calculate the fuel consumption rate and air–fuel moisture ratios, respectively.

### 2.6. Uncertainty Analysis

The results from proximate analyses, biodiesel production, fuel properties and engine characteristics were analyzed for any uncertainty in their data in order to ensure their accuracy. In addition, this type of analysis addresses the variation in results that arise due to natural phenomena and human or technical errors. For the purpose of ensuring accuracy in experimental results, each experiment was conducted in triplicates, and their resultant values were reported in terms of mean values ± standard deviation as calculated using Equation (2) [36,37]. Moreover, these deviations have been signified in the graphical plots representing the experimental results, wherever applicable.

(2) $$\sigma =\sqrt{\frac{\sum {\left(x_{i}-\mu \right)}^{2}}{N}}$$

Here, σ = standard deviation of analyzed population, *N* = size of the analyzed population, *x_i_* = value from population and *μ* = mean value of analyzed results.

## 3. Results and Discussion

### 3.1. Proximate Composition of Collected Animal Wastes

**Figure 3:**
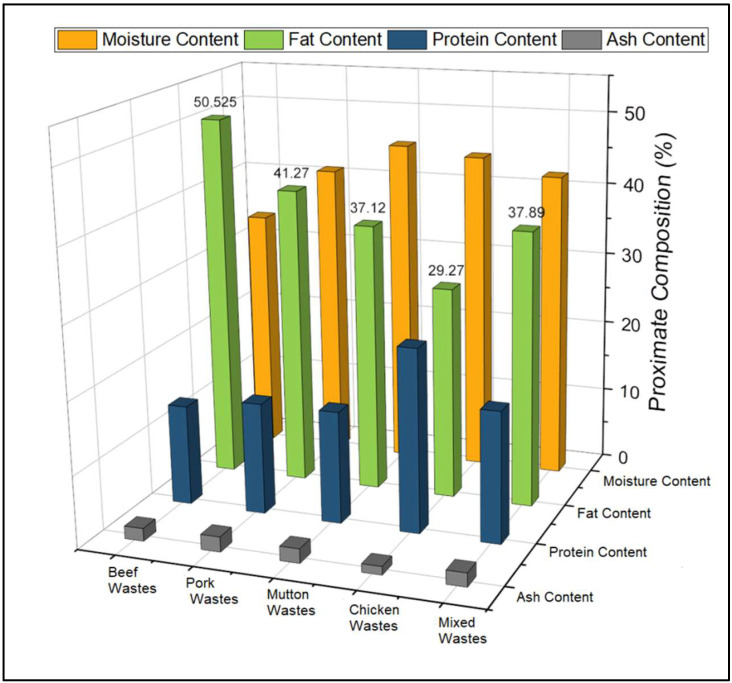
Proximate composition of collected animal wastes.

From the proximate analyses (Figure 3), the composition of each raw animal waste was found to be as follows: beef (moisture—33.81 ± 1.5%, fat—50.53 ± 2.41%, protein—13.9 ± 1.22%, and ash—1.77 ± 0.45%), pork (moisture—41.23 ± 1.44%, fat—41.27 ± 1.67%, protein—15.41 ± 0.9%, and ash—2.09 ± 0.75%), mutton (moisture—45.43 ± 1.66%, fat—37.12 ± 1.94%, protein—15.41 ± 0.57%, and ash—1.98 ± 0.61%), and chicken (moisture—44.36 ± 2.27%, fat—29.27 ± 1.19%, protein—25.21 ± 0.85%, and ash- 1.16 ± 0.45%). Moreover, the proximate composition of mixed raw animal wastes (in equivalent ratio) was determined to be moisture—42.24 ± 1.63%, fat—37.89 ± 1.26%, protein—17.98 ± 1.03%, and Ash—1.89 ± 0.67%. Increased fat content was contributed by beef wastes and pork wastes due to their nature and food habitat [38,39]. Specifically, a large portion of these fats was accumulated in the subcutaneous wastes, while other portions of these fats were contributed from the intramuscular wastes. Waste oil was recovered exclusively from these intramuscular waste fractions. Moreover, the dry rendering technique was foreseen as the most ideal technique for rendering these fat and oil wastes, accounting for their higher rate of rendering efficiency [40,41].

### 3.2. Fatty Acid Composition of Rendered WAF-O

Looking into the FA composition of the rendered WAF-O, oleic acid (39.68%), palmitic acid (28.33%) and stearic acid (13.19%) were identified as dominant FAs with an overall saturated and unsaturated content of 46.54% and 53.46%, respectively. Increased concentrations of oleic acid were contributed by the rendered beef tallow and lard, whereas linoleic acid was contributed by lard and chicken fat. On the other hand, palmitic acid and stearic acid were distributed from suet and tallow and were responsible for increasing the overall saturation content in the resultant WAF-O. Ethyl oleate (38.25%), ethyl palmitate (30.62%) and ethyl stearate (14.27%) were identified as the dominant FAEs available in the resultant WAF-O biodiesel with an overall saturated and unsaturated content of 50.6% and 49.4%, respectively. Unfortunately, a reduction in the overall unsaturated content in the resultant WAF-O biodiesel was due to low reactivity rates between ethanol and unsaturated FAs in the WAF-O.

### 3.3. Optimization of Transterifcation of Rendered Fat-Oil (OFAT Method)

**Figure 4:**
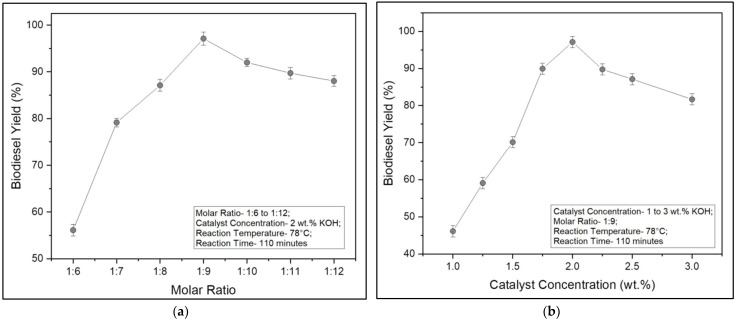

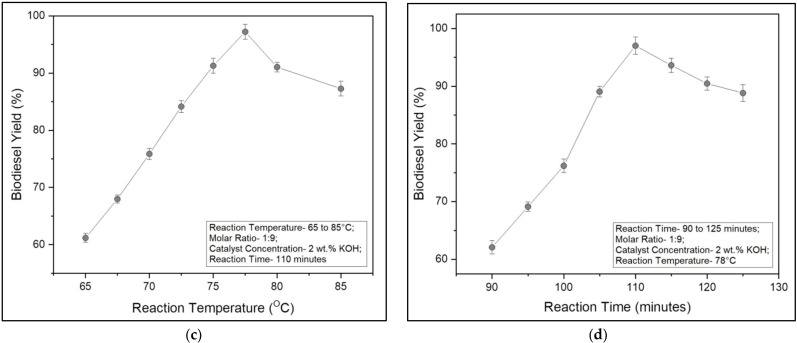
(**a**) Biodiesel yield for varying molar ratios; (**b**) biodiesel yield for varying catalyst concentrations; (**c**) biodiesel yield for varying reaction temperatures; (**d**) biodiesel yield for varying reaction times.

As mentioned earlier, the rendered WAF-O was converted into biodiesel through the means of base-catalyzed transesterification using ethanol as the solvent and KOH as the ideal homogeneous base catalyst. To begin with, ethanol was chosen over other solvents in view of its increased renewability and its contribution to improving the overall fuel properties of the resultant biodiesel. Furthermore, introducing ethanol into the reaction allowed the system to be operated at higher temperatures (75–80 °C), which helped enhance the overall reaction rate and yield and also ensured a higher rate of solubility of WAF-O in the reaction mix [42]. Meanwhile, KOH was chosen for its successful ability to transesterify WAF-O into biodiesel compared to NaOH. In addition, KOH played a less significant role in the saponification of WAF-O [43]. Figure 4a–d plots the biodiesel yields for varying reaction parameters using the OFAT technique. The optimized reaction parameters for transesterifying the WAF-O into its biodiesel were as follows: molar ratio—1:9, catalyst concentration—2 wt.% of KOH, reaction temperature—78 °C, and reaction time—110 min. Here, the elevated molar ratio and catalyst concentration were explained by the long-chain FAs available in the triglycerides of WAF-O, which required large quantities of solvent and catalyst to undergo a complete conversion. Likewise, elevated reaction temperature and prolonged reaction time were attributed to chemical changes such as the weakening of Van der Waals forces of attraction of FA ions and enhancing the affinity for strong nucleophilic ethyl ions [44]. Table 2 summarizes the factors influencing the biodiesel yield for different ranges of reaction parameters.

### 3.4. Fuel Properties of WAF-O Biodiesel

The produced WAF-O biodiesel was tested for its fuel properties as per ASTM standards (Table 3), and the values obtained were in close agreement with the fuel properties of petroleum diesel. The density of WAF-O biodiesel was found to be 880.93 ± 2.7 kg/m^3^ and was 6.14% greater than the density of neat diesel. Likewise, the kinematic viscosity of biodiesel was found to be 4.85 ± 0.16 mm^2^/s and was 34.72% greater than the viscosity of neat diesel. Higher rates of density and viscosity for WAF-O biodiesel were accounted for by the long-chain FAEs in the WAF-O biodiesel. Increased flash point (164 °C) and slightly reduced cloud point (8.5 °C) and pour point (1 °C) were attributed to the long-chain FAEs in the WAF-O biodiesel that collectively ensured the WAF-O biodiesel’s safe handling. Furthermore, saturated FAEs contributed to the higher CN of the WAF-O biodiesel, which was found to be greater by 24% than neat diesel. In addition, these FAEs also played a significant role in providing reduced ignition delays (ID). The CV of WAF-O biodiesel was found to be 38.89 ± 0.8 MJ/kg, which was 8.49% lesser than neat diesel and ranged in between the CV of other individual fat biodiesel, such as tallow biodiesel and chicken fat biodiesel [48]. The molecular formula of WAF-O biodiesel was calculated as C_19_H_37_O_2_, with carbon molecules contributing up to 76.71% content, while hydrogen and oxygen contributed 12.53% and 10.76%, respectively. Based on the molecular formula, it was concluded that the resultant WAF-O biodiesel was slightly unsaturated, and the average length of a carbon chain in a FA moiety was 17, which signified the major contribution of the characterized dominant FAs in the WAF-O biodiesel. More emphatically, the presence of oxygen in its molecular structure confirmed the produced WAF-O biodiesel as an oxygenated fuel.

**Figure 5:**
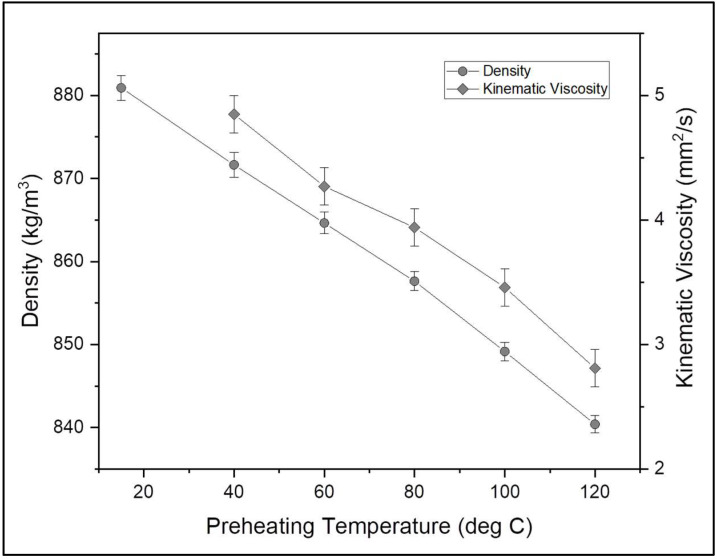
Variation in density and kinematic viscosity of WAF-O biodiesel at varying temperatures.

In order to understand the effect of preheating on WAF-O biodiesel, temperature-dependent fuel properties, such as density and viscosity, were recorded for the WAF-O biodiesel preheated at different temperatures (60, 80, 100 and 120 °C). As a result, both density and viscosity of WAF-O biodiesel were reduced by 0.383 kg/m^3^ and 0.025 mm^2^/s for every 1 °C rise in preheating temperature. This reaction was explained by the weakening of intermolecular forces between the molecules present in the WAF-O biodiesel upon heating and was also reported for other oil- and fat-based biodiesel [28,49]. Figure 5 represents the graphical plot of density and kinematic viscosity for varying temperatures.

### 3.5. Engine Characteristics of WAF-O Biodiesel

#### 3.5.1. Combustion Characteristics

##### In-Cylinder Pressure

**Figure 6:**
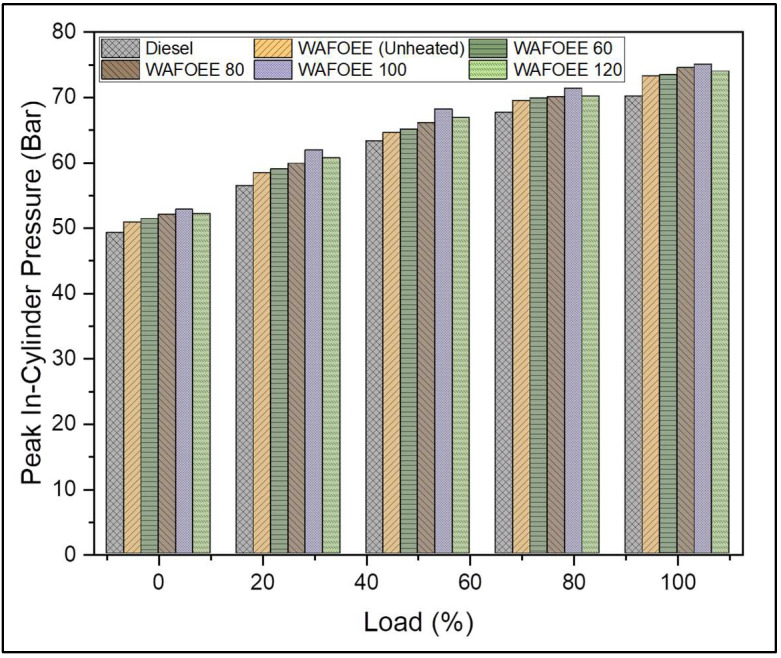
Peak in-cylinder pressure of neat diesel and biodiesel samples for varying engine loads.

The pressure developed inside the cylinder signifies the degree of homogeneous mixing between the injected fuel and air and plays a vital role in enhancing the rate of combustion. In fact, the pressure inside the cylinder increases with load due to the combustion of the large amounts of fuel injected in order to meet the increasing energy demand. A similar trend (Figure 6) was reported in this present study and was backed up by the comparable results reported while studying the combustion characteristics of preheated oil- and fat-based biodiesel that reported a rise in cylinder pressure of 5–10% [21,30]. Biodiesel samples exhibited higher mean and peak in-cylinder pressures compared with neat diesel in regard to their higher CN and fuel-bound oxygen content [50,51]. Supporting this, peak in-cylinder pressure of diesel was found to be 3.08% less than neat biodiesel and 3.76%, 4.87%, 6.83% and 5.28% less than WAFOEE 60, WAFOEE 80, WAFOEE 100 and WAFOEE 120 samples, respectively. Further, peak in-cylinder pressures of preheated biodiesel were found to be higher than unheated WAF-O biodiesel by 0.7%, 1.88%, 4.03%, and 2.33% for WAFOEE 60, WAFOEE 80, WAFOEE 100 and WAFOEE 120 samples, respectively. Above all, the highest peak in- cylinder pressure was recorded as 75.02 ± 1.36 Bar at 7 °CA for a WAFOEE 100 sample. These data are attributed to the preheating of biodiesel samples that reduced their viscosities, leading to enhanced fuel atomization and the early start of combustion. In addition, atomization allows the WAF-O biodiesel samples to combust rapidly using their fuel-bound oxygen present in their molecular structure. In contrast, the WAFOEE 120 sample failed to register an optimal peak in-cylinder pressure because of its very low viscosity, causing the fuel to combust poorly due to a very early start of the combustion reaction. Moreover, no knocking activities were noted for any samples throughout the experimental runs.

##### Heat Release Rate

HRR curves represent the distribution of heat inside an engine cylinder given in terms of energy [52]. HRR signifies the initiation and completion of combustion and is calculated from the recorded in-cylinder pressures using the first law of thermodynamics, assuming the combustion is taking place inside a closed system. Equation (3) represents the mathematical correlation used for calculating the HRR, taking in-cylinder pressure as an independent variable [53,54].

(3) $$\frac{dQ}{d\theta }=\frac{k}{k-1}P\frac{dV}{d\theta }+\frac{1}{k-1}V\frac{dP}{d\theta }$$

Instantaneous HRRs (IHRRs) of biodiesel samples were found to be greater than neat diesel fuel in view of their higher fuel-bound oxygen content and CN than compared to neat diesel [35,55]. In contrast, neat diesel reported the highest cumulative HRR (CHRR) compared to biodiesel samples, which was explained by its prolonged ID and rapid combustion of accumulated fuel. Relatively, unheated WAF-O biodiesel exhibits its IHRR at 4.93% greater than the diesel sample; however, preheated samples (WAFOEE 60, WAFOEE 80, WAFOEE 100 and WAFOEE 120 samples) reported higher IHRR by 0.81%, 2.44%, 4.36%, 1.84%, respectively. The highest IHRR (77.77 ± 2.69 KJ/m^3^.deg) for WAFOEE 100 biodiesel was due to its reduced viscosity, which enhanced its atomization and allowed for its early start of combustion. As a result, a homogeneously mixed air–fuel blend combusted rapidly using the fuel-bound oxygen content within it. Moreover, the diesel sample exhibited higher CHRR following its prolonged ID due to low CN, which produced a higher amount of heat during the later stages of combustion stroke. This was a result of the rapid combustion of highly atomized accumulated fuel [56]. Likewise, literature focusing on preheating oil- and fat-based biodiesel reported heat release rates similar to the rates measured in this present study and were explained by the reduced viscosity and early start of ignition along with their oxygen content [21,30]. The highest CHRR was reported for neat diesel at its full load condition, followed by other biodiesel samples and was measured at 1624.4 ± 3.27 kJ/m^3^.

##### Ignition Delay

**Figure 7:**
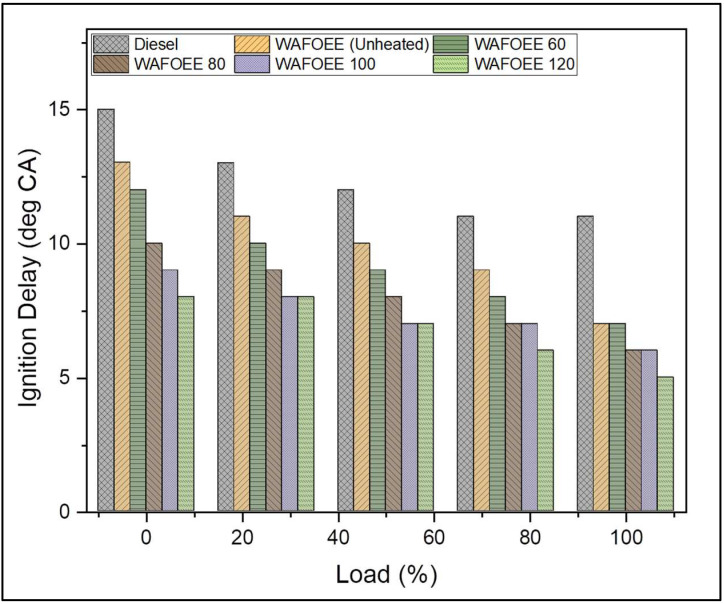
Ignition delay of neat diesel and biodiesel samples for varying engine loads.

ID talks about the delay period between the start of fuel injection (SOI) and start of combustion (SOC) and was calculated from the IHRR and in-cylinder pressure in terms of their corresponding crank angles. In general, the ID window is smaller for biodiesel fuel on account of its high CN, which favors the early start of combustion compared to neat diesel. Based on combustion data, SOI was reported to occur at 20 °CA bTDC (before top dead center—position of engine piston) throughout the experimental runs for all samples. As mentioned earlier, all biodiesel samples exhibited reduced ID due to higher CN contributed by their long-chain FAEs. In addition, ID was also reduced with the rise in preheating temperatures. Supporting this, unheated biodiesel exhibited a reduction in ID by 2.4 °CA (on average) compared to neat diesel. Meanwhile, preheating of WAF-O biodiesel reduced its average ID by 0.8, 2, 2.6, 3.2 °CA for WAFOEE 60, WAFOEE 80, WAFOEE 100 and WAFOEE 120 samples, respectively, compared to unheated samples. This may be due to their reduced viscosities along with their high CN that, in turn, advanced their SOC [57,58,59]. Figure 7 represents the graphical plot for the ID of diesel and biodiesel samples.

##### Exhaust Gas Temperature

**Figure 8:**
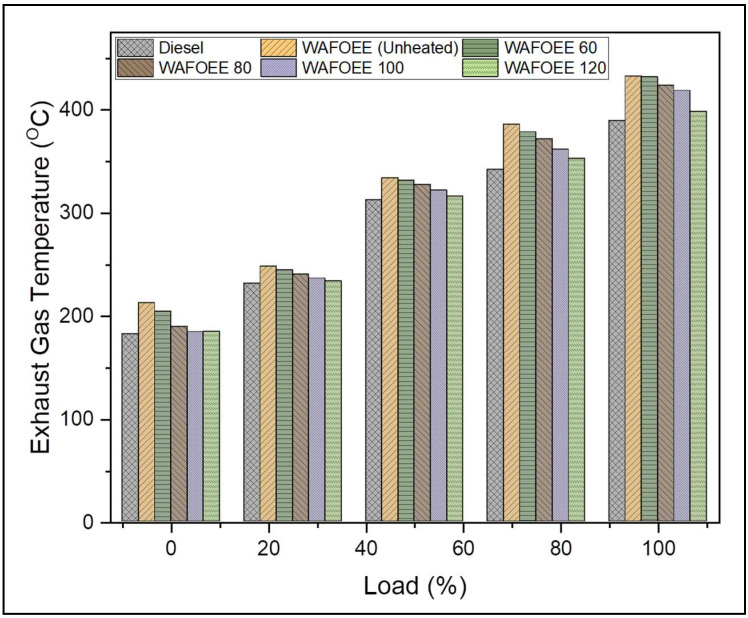
Exhaust gas temperature of neat diesel and biodiesel samples for varying engine loads.

Exhaust gas temperature (EGT) indicates the complete combustion of fuel inside the cylinder and is an effective indicator of the engine’s healthy performance. Primarily, it is regulated by both the engine’s operating condition and properties of the fuel used and plays a crucial role in deciding the NO_X_ emissions in exhaust gas [54]. Any fuel with low CN exhibits prolonged ID followed by longer combustion duration that tends to report higher EGT and NO_X_ emissions. In contrast, the EGT of neat diesel was found to be 9.59% lower than unheated WAF-O biodiesel and remained reduced by 8.33%, 6.08%, 4.25% and 1.87% for WAFOEE 60, WAFOEE 80, WAFOEE 100 and WAFOEE 120 samples, respectively. Biodiesel reports higher EGTs than compared to neat diesel due to its shorter ID attributed to its higher CN, which provides sufficient time to undergo complete combustion using the fuel-bound oxygen content. Upon comparing the biodiesel samples, preheated samples reported reduced EGT (1.38%, 3.74%, 5.58%, 7.87% for WAFOEE 60, WAFOEE 80, WAFOEE 100 and WAFOEE 120 samples, respectively) compared to unheated biodiesel. Lower EGT thus occurs via reduced viscosity of the preheated biodiesel samples, which results in a higher degree of atomization and shortened ID. In fact, a slight reduction in the adiabatic flame temperature eventually reduced both EGT and NOx emissions [60]. In spite of an early SOC, slightly elevated EGTs were reported for preheated samples due to the presence of fuel-bound oxygen in them, which ensured their complete oxidation. Supporting this, Bayrakceken (2011) reported a reduction in EGT while increasing the preheating temperature for animal tallow biodiesel and also hinted at the reduction in the concentration of NOx emissions [30]. Figure 8 signifies the graphical plot for EGT of diesel and biodiesel samples.

#### 3.5.2. Performance Characteristics

##### Specific Fuel Consumption and Brake Thermal Efficiency

**Figure 9:**
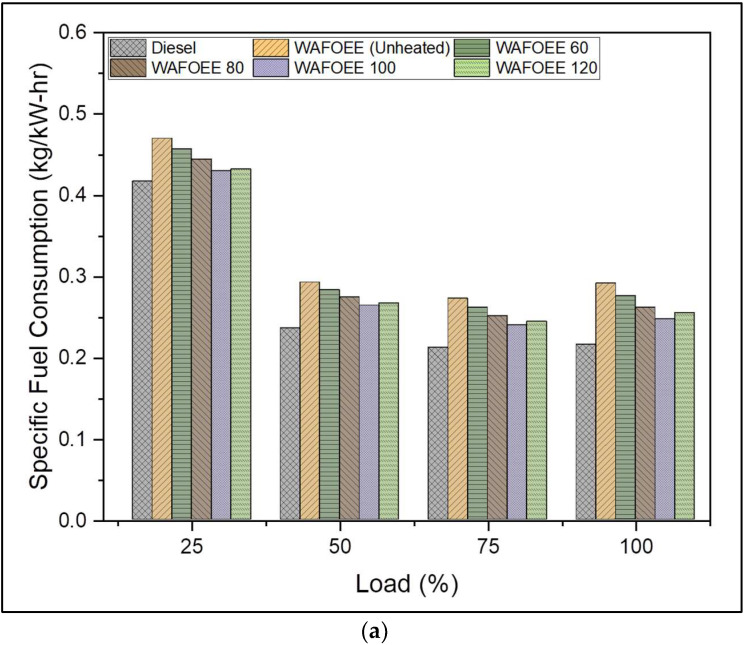

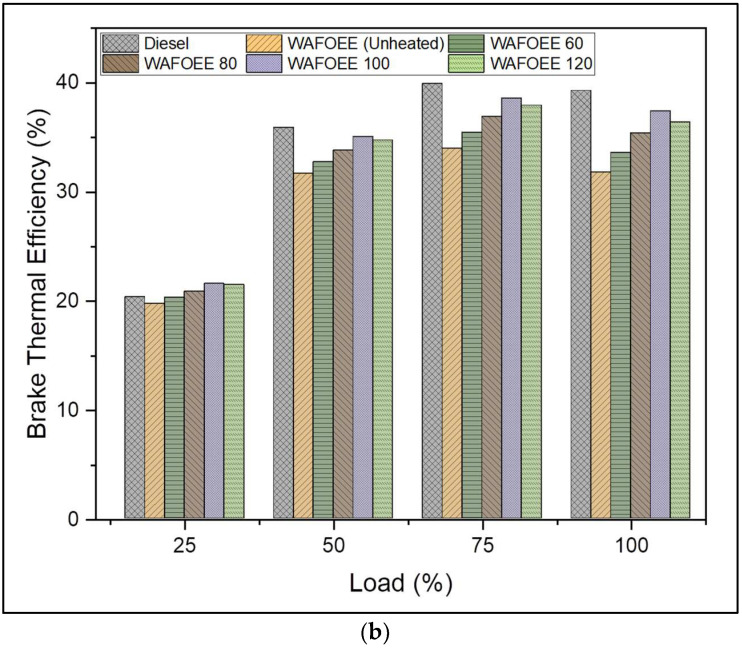
(**a**) Specific fuel consumption of neat diesel and biodiesel samples for varying engine loads. (**b**) Brake thermal efficiency of neat diesel and biodiesel samples for varying engine loads.

For the purpose of understanding the performance of preheated WAF-O biodiesel in CI engines, data related to specific fuel consumption (SFC) and brake thermal efficiencies (BTE) were studied in detail. SFC quantifies the volume of the fuel required for combustion in order to generate an output power of 1 unit. SFC conversely decreases with increasing loads in view of its increasing brake power [61]. On the other hand, BTE is the effectiveness (given as a percentage) showcased by an engine when converting the stored chemical energy of fuel into actual mechanical work. Unlike SFC, BTE increases with an increase in load in order to meet the increased energy demand by consuming a large quantity of fuel. The SFC of neat diesel was calculated to be 18.45% less than unheated WAF-O biodiesel. Subsequently, this reduced SFC rate resulted in increased BTE for neat diesel by 15.53% due to its low viscosity and superior CV [62]. However, preheating the WAF-O biodiesel reduced its SFC by 3.71%, 7.15%, 10.89% and 9.69% compared to unheated biodiesel, giving rise to increased BTE by 4.13%, 8.31%, 13.15% and 11.35% (for WAFOEE 60, WAFOEE 80, WAFOEE 100 and WAFOEE 120 samples, respectively). Explaining this, preheating of WAF-O biodiesel very likely reduced its density and viscosity relative to the preheating temperature, thus ensuring the injection of adequate amounts of fuel with proper atomization inside the cylinder. This allowed early SOC, followed by complete combustion of the injected fuel, which extracted the maximum energy output from it. In fact, a similar trend in BTE was reported upon preheating other oil- and fat-based biodiesel, with an increase in BTE ranging between 9 and 18%, and was explained by enhanced flow characteristics of the test samples [21,28,30]. However, poor BTE for the WAFOEE 120 sample is explained by its inability to properly combust due to poor viscosity and increased fuel consumption to produce the equivalent amount of energy output. Figure 9a,b shows the SFC and BTE plots of neat diesel and biodiesel samples.

#### 3.5.3. Emission Characteristics

##### Carbon Monoxide and Carbon Dioxide Emissions

Generally, combustion of any hydrocarbon results in the formation of carbon dioxide (CO_2_) in the case of complete combustion or carbon monoxide (CO) in the case of incomplete combustion. The formation of these gases is entirely dependent on the properties of fuel used, such as degree of unsaturation, carbon to hydrogen ratio and presence of aromaticity. Other factors influencing the formation of CO emissions include ineffective engine operating conditions resulting in improper atomization, limited time duration for homogeneous mixing and proper combustion, and most importantly, an inappropriate air (oxygen) to fuel ratio [63,64].

**Figure 10:**
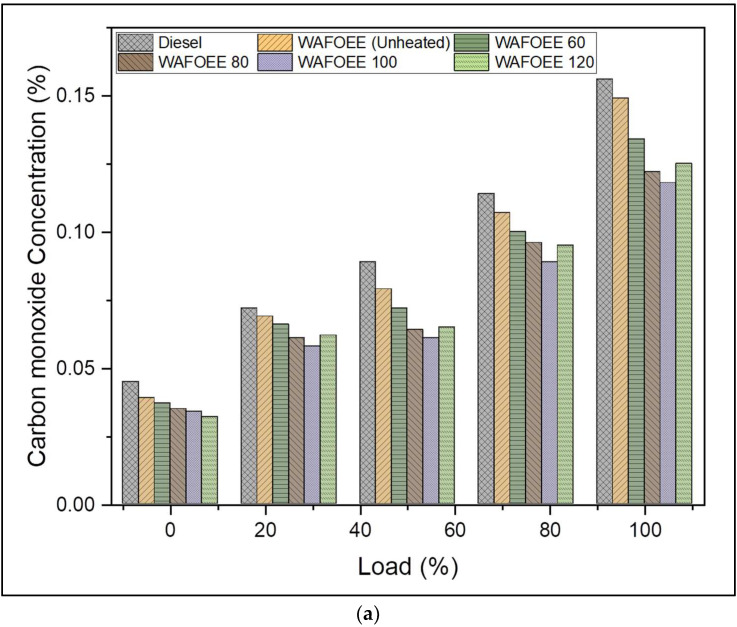

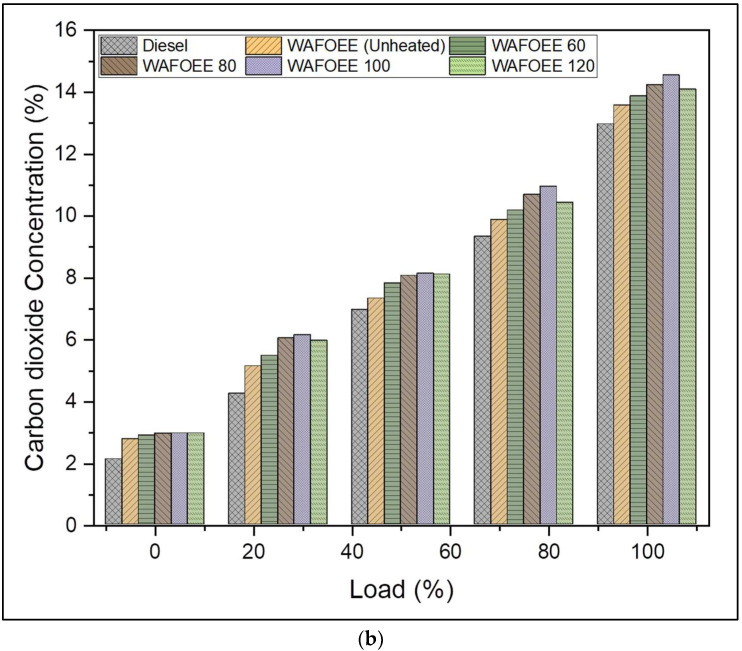
(**a**) CO emissions of neat diesel and biodiesel samples for varying engine loads; (**b**) CO_2_ emissions of neat diesel and biodiesel samples for varying engine loads.

In general, biodiesel samples exhibit low CO and high CO_2_ emissions compared with neat diesel, citing their fuel-bound oxygen content, which ensures its complete oxidation during combustion. In this case, CO emission of unheated biodiesel was reduced only by 6.93%, while preheated samples registered emissions reduced by as much as 14.08%, 20.59%, 24.37% and 20.38% (WAFOEE 60, WAFOEE 80, WAFOEE 100 and WAFOEE 120 samples, respectively). Both unheated and preheated biodiesel demonstrated that augmented CO_2_ emissions and unheated biodiesel expressed emissions increased by 8.6%. Emissions of preheated samples (WAFOEE 60, WAFOEE 80, WAFOEE 100 and WAFOEE 120) increased by 12.92%, 17.81%, 19.94% and 16.63%, respectively. Meanwhile, preheated samples reported reduced CO emission and increased CO_2_ emission compared to both plain diesel and unheated WAF-O biodiesel. Again, reduced viscosity allowed the preheated samples to atomize homogeneously and combust completely, in addition to their shortened ID. Compared to unheated biodiesel, CO emissions of preheated samples were reduced by 7.67%, 14.67%, 18.74 and 14.45% (WAFOEE 60, WAFOEE 80, WAFOEE 100 and WAFOEE 120 samples, respectively), while their CO_2_ emissions increased by 3.98%, 8.48%, 10.45% and 7.4% (WAFOEE 60, WAFOEE 80, WAFOEE 100 and WAFOEE 120 samples, respectively). Supporting this, Bayrakceken (2011) reported a reduction in CO emission of preheated tallow biodiesel at 100 °C as 45.77% [30]; however, Kodate et al., 2020, only reported a 10% reduction in CO emission for preheated Karanja oil biodiesel [21], thereby acknowledging the reported CO emission trend from this present study. Moreover, WAFOEE 120 biodiesel reported increased CO and reduced CO_2_ emissions on account of its poor rate of combustion due to reduced viscosity and increased ability to rapidly initiate combustion, resulting in improper oxidation of long-chain FAEs [35,56]. Furthermore, a rise in CO and CO_2_ emissions for both neat diesel and biodiesel occurred with increasing load, signifying the consumption of an increased amount of fuel to meet the required energy demand. Figure 10a,b represents the graphical plot of CO and CO_2_ emissions for neat diesel and biodiesel samples, respectively.

##### Nitrogen Oxides and Hydrocarbon Emissions

Nitrogen oxide (NO_X_) emission is a temperature-dependent phenomenon that can be regarded as the indicator of elevated EGTs produced during proper and complete combustion inside the cylinder. NO_X_ emission is widely influenced by ID and combustion duration, fuel-bound oxygen content and viscosity of the fuel. Most of the time, biodiesel exhibits higher NO_X_ emissions than neat diesel due to higher CN, which shortens the ID, thereby providing sufficient time to undergo complete combustion using fuel-bound oxygen content [6]. Likewise, NO_X_ emissions were reported to be 14.39%, 10.05%, 7.19% and 2.06% higher for preheated samples (WAFOEE 60, WAFOEE 80, WAFOEE 100 and WAFOEE 120 samples, respectively) and even higher for unheated biodiesel (16.84%). Compared to biodiesel samples, preheated samples reported reduced NO_X_ emissions (2.1%, 5.81%, 8.26%, and 12.66% for WAFOEE 60, WAFOEE 80, WAFOEE 100 and WAFOEE 120 samples, respectively) compared to unheated biodiesel. Eventually, preheating biodiesel reduced both premixed combustion duration and overall combustion duration, followed by slightly lowered adiabatic flame temperatures compared to its unheated counterpart [35,60]. In fact, similar reduction in concentration of NOx emission with a rise in the preheating temperature was noted upon studying animal tallow biodiesel [30]. It is worth mentioning that this present study followed the Zeldovich (Thermal NO_X_) mechanism for its NOx formation, citing the involvement of a lean fuel mixture due to preheating [1,65].

**Figure 11:**
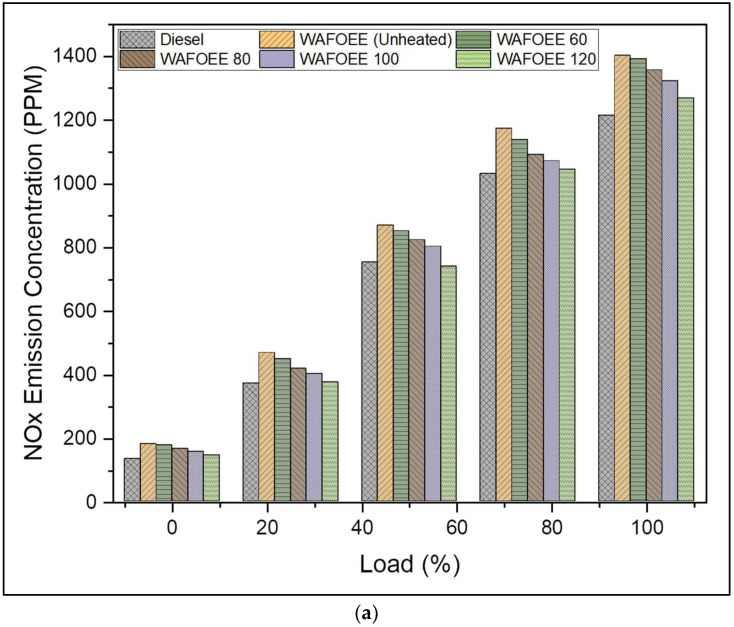

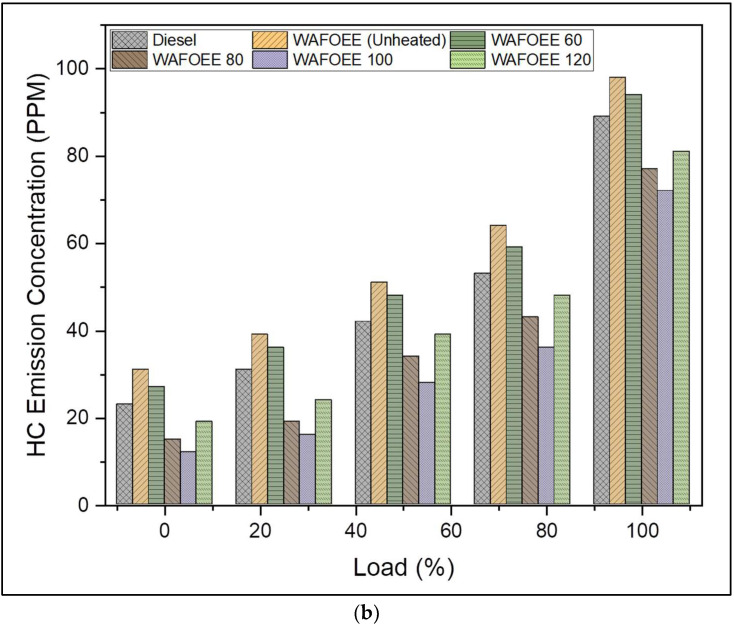
(**a**) NOX emissions of neat diesel and biodiesel samples for varying engine loads. (**b**) HC emissions of neat diesel and biodiesel samples for varying engine loads.

As far as hydrocarbon (HC) emissions are concerned, they mainly arise due to the inability of the atomized fuel to become completely combusted in a local region near the cylinder wall due to low regional temperature. In addition, a poor kinetic rate of chemical combustion and a quenched flame have also been identified as root causes for these HC emissions [66]. Here, the HC emissions of neat diesel were in the range of the HC emissions of biodiesel samples. Meanwhile, lower concentrations of HC emissions were reported for biodiesel samples preheated at high temperatures, whereas samples preheated at low temperatures reported increased concentrations [50]. These phenomena were attributed to the reduced viscosity of preheated samples, which ensured homogeneous atomization and vaporization, which allowed proper air–fuel mixing to undergo complete combustion. As a result, HC emissions of WAFOEE 100 and WAFOEE 120 samples were found to be 31.09 and 11.34% lower than neat diesel, whereas WAF-O biodiesel and WAFOEE 60 exhibited higher HC emissions compared to the diesel sample. All preheated samples displayed very low HC emissions signifying that preheating biodiesel prior to combustion favored proper atomization and complete oxidation of fuel. As a matter of fact, numerous studies have claimed reduced HC emissions for both unheated and preheated biodiesel, with the latter reporting a higher reduction rate resembling the HC emission reported in this present study [21,28,30,67]. Moreover, HC emissions increased with load conditions for both sample types due to increased fuel consumption. In addition, HC emissions remained lower for biodiesel samples in view of their high in-cylinder pressures and temperatures, apart from their fuel-bound oxygen [68]. Figure 11a,b represents the graphical plot of NOx and HC emissions for neat diesel and biodiesel samples, respectively.

## 4. Conclusions

Thus, the experimental study investigating the effects of fuel preheating on engine characteristics of waste animal fat-oil (WAF-O) biodiesel in a single-cylinder CI engine was carried out successfully, and some of the majorly drawn conclusions are as follows:

1. WAF-O was identified as the ideal feedstock for producing high energy density biodiesel, with oleic acid (38.25%), palmitic acid (30.62%) and stearic acid (14.27%) being identified as its dominant FAs and their corresponding esters, and it was fairly evident that natural fats and oil are predominantly distributed with oleic acid and palmitic acid.
2. Ethanol-based transesterification using KOH as the homogeneous base catalyst was found to be highly effective at producing WAF-O biodiesel, with their high molar ratio and catalyst concentration explained by the long-chain FAs available in the WAF-O samples.
3. WAF-O biodiesel reported slightly higher density, viscosity, flash point and CN pertaining to long-chain FAEs and reduced CVs due to the absence of sulfur. In addition, preheating of WAF-O biodiesel weakened the intermolecular forces of attraction between its molecules and resulted in reduced density and kinematic viscosity, thereby leading to a reduced flash point and ID.
4. Preheated WAF-O biodiesel samples reported enhanced combustion (in-cylinder pressure increased by 2.24%) and performance characteristics (BTE increased by 9.23%) in view of their improvised atomization and vaporization from fuel preheating. However, higher preheating temperatures (>120 °C) resulted in poor performance of the WAF-O biodiesel, especially when it resulted in increased fuel consumption.
5. Preheating of WAF-O biodiesel samples resulted in reduced CO and HC (13.88% and 26.94%, respectively) and increased CO_2_ emissions due to complete oxidation of fuel during combustion. This was acknowledged by the reduced viscosity and increased CN, which provided sufficient time to complete the combustion of the samples.
6. Preheated biodiesel samples registered a slight reduction in EGTs and NOx compared to unheated biodiesel due to their slightly reduced combustion durations and low adiabatic flame temperatures inside the cylinder. Higher values of EGTs and NOx can be explained by the presence of long-chain FAEs available for reactions.

This study strongly recommends the preheating of biodiesel as an effective technique for improving its engine characteristics, especially for large-scale and commercial applications. The preheating of the fuel can be achieved by augmenting the heat exchanging system with the engine’s waste heat recovery system or exhaust gas heat recovery system, which can be studied for further enhancement in efficiency. Ultimately, this modification in engine setup and fuel properties will tend to enhance the overall performance of both the engine and fuel. This will eventually reduce the emissions level drastically, thereby paving the way for the effective running of the engine in a sustainable manner. In addition to preheating the biodiesel, attempts such as preheating the inlet air and using it for combusting unheated/preheated biodiesel can also be studied, thus enabling one to utilize the maximum available energy in a renewable way to meet the global power demand more sustainably.

## Figures and Tables

**Table 1:** Technical specifications of Kirloskar TV1 Engine and AVL DI GAS 444 N Flue Gas Analyzer [35].

Kirloskar Engine TV 1 Specifications		AVL DI GAS 444 N (Five Gas Analyser)	
Type: Four Stroke, Single Cylinder Water Cooled		Measurement	Resolution
Rated Power	5.2 KW	CO (0–15% Vol)	0.0001% Vol
Rated Speed	1500 rpm	HC (0–20,000 ppm Vol)	1 ppm/10 ppm
Bore diameter (D)	87.5 mm	CO_2_ (0–20% Vol)	0.1% Vol
Stroke (L)	110 mm	O_2_ (0–25% Vol)	0.01% Vol
Compression Ratio	17.5:1	NO_X_ (0–6000 ppm Vol)	1 ppm Vol

**Table 2:** Factors influencing the biodiesel yield for reaction parameters.

Reaction Parameter	Minima	Maxima	Optimized Value	Range	Biodiesel Yield	Remarks
Molar Ratio	1:6	1:12	1:9	1:6–1:9	Increased	Availability of a sufficient amount of ethanol for completing the transesterification of WAF-O [4,45].
				1:9–1:12	Decreased	Excess ethanol available in the reaction system helped in recombining the produced esters with glycerol to form back monoglycerides. Excess ethanol diluted the glycerol, which was carried away with biodiesel and complicated the separation, resulting in reduced biodiesel yield.
Catalyst Concentration	1 wt.%	3 wt.%	2 wt.%	1–2	Increased	Sufficient availability of KOH that produced an adequate number of O_2_^−^ active sites used for extracting H^+^ ions from the ethanol, leaving behind C_2_H_5_O^−^ ions [46].
				2–3	Decreased	Slightly higher concentration of KOH favored the recombination of synthesized FAEs and glycerol into monoglycerides. Excess KOH led to soap formation due to the saponification of monoglycerides.
Reaction Temperature	65 °C	85 °C	78 °C	65–78	Increased	Maintained the liquefied state of WAF-O and enhanced interactions with ethanol, improving both conversion rate and biodiesel yield.
				78–85	Decreased	Reduced the availability of ethanol due to its evaporation, resulting in its deficiency [47].
Reaction Time	90 min.	125 min.	110 min.	90–110	Increased	Required necessary time for inducing chemical changes such as weakening van der Waals force of attraction of FA ions and enhancing the affinity for strong nucleophilic ethyl ions [44,45].
				110–125	Decreased	Favored recombination of resultant FAEs with glycerol to form monoglycerides, thereby reducing the biodiesel yield.

O_2_^−^: oxygen ion; H^+^: hydrogen ion; C_2_H_5_O^−^: ethyl ion.

**Table 3:** Fuel properties of WAF-O biodiesel and neat diesel.

Fuel Property	WAF-O Biodiesel	Neat Diesel	Permissible Range	Unit	ASTM Standard
Density	880.93 ± 2.7	830	-	kg/m^3^	ASTM D1298
Specific Gravity	0.871	0.83	0.86–0.90	-	ASTM D1298
Kinematic Viscosity	4.85 ± 0.16	3.7	1.9–6.0	mm^2^/s	ASTM D445
Cetane Number	62 ± 0.5	50	47 (min)	-	ASTM D613
Calorific Value	38.89 ± 0.8	42.5	35 to 43	MJ/Kg	ASTM D240
Flash Point	164 ± 1.43	75	130 (min)	°C	ASTM D93-16
Fire Point	176 ± 1.75	86	-	°C	ASTM D93-16
Cloud Point	8.5 ± 1.35	0	−3 to 12	°C	ASTM D2500
Pour Point	1 ± 0.52	−13	−15 to 10	°C	ASTM D7346-15
Acid Number	0.26 ± 0.01	0.07	0.8 (max)	mg KOH/g fat	ASTM D664
Free Fatty Acid	0.13 ± 0.01	0.03	-	%	ASTM D664
Saponification Number	187.88 ± 0.76	-	-	mg KOH/g fat	ASTM D5558
Iodine Value	48.56 ± 0.96	-	120 (max)	g I_2_/100 gm	ASTM D5554
Molecular Formula	C_19_H_37_O_2_	C_16_H_28_	-	-	-
Molecular Weight	297.5	220.39	-	g/mol	-
Carbon	76.71 ± 0.37	85.16	-	wt.%	ASTM D5291
Hydrogen	12.54 ± 0.56	14.26	-	wt.%	ASTM D5291
Oxygen	10.76 ± 0.44	-	-	wt.%	ASTM D5291
Sulfur	0.0001	0.152	-	wt.%	ASTM D5453
Phosphorus	0.0001	-	-	wt.%	ASTM D4951

## Data Availability

All relevant data are included within the article.

## Abbreviations

AOAC	Association of Official Analytical Chemists
ASTM	American Society for Testing and Materials
BTE	Brake Thermal Efficiency
CHRR	Cumulative Heat Release Rate
CI	Compression Ignition
CN	Cetane Number
CO	Carbon monoxide
CO2	Carbon dioxide
CV	Calorific Value
EGT	Exhaust Gas Temperature
FAs	Fatty Acids
FAEs	Fatty Acid Esters
GC-MS	Gas Chromatography and Mass Spectrometry
HC	Hydrocarbon
HRR	Heat Release Rate
ID	Ignition Delay
IHRR	Instantaneous Heat Release Rate
KOH	Potassium Hydroxide
NOx	Nitrous Oxide
OFAT	One Factor at a Time
SFC	Specific Fuel Consumption
SFO	Stone Fruit Kernel Oil
WAF-O	Waste Animal Fat Oil Biodiesel
WAFOEE	Waste Animal Fat Oil Ethyl Ester Biodiesel
WAFOEE 60	Waste Animal Fat Oil Ethyl Ester heated at 60 °C
WAFOEE 80	Waste Animal Fat Oil Ethyl Ester heated at 80 °C
WAFOEE 100	Waste Animal Fat Oil Ethyl Ester heated at 100 °C
WAFOEE 120	Waste Animal Fat Oil Ethyl Ester heated at 120 °C
